# The global environmental injustice of fast fashion

**DOI:** 10.1186/s12940-018-0433-7

**Published:** 2018-12-27

**Authors:** Rachel Bick, Erika Halsey, Christine C. Ekenga

**Affiliations:** Brown School, Washington University in St. Louis, Campus Box 1196, One Brookings Drive, St. Louis, MO 63130 USA

**Keywords:** Environmental health, Occupational health, Global health, Environmental justice, Sustainability, Fast fashion

## Abstract

Fast fashion, inexpensive and widely available of-the-moment garments, has changed the way people buy and dispose of clothing. By selling large quantities of clothing at cheap prices, fast fashion has emerged as a dominant business model, causing garment consumption to skyrocket. While this transition is sometimes heralded as the “democratization” of fashion in which the latest styles are available to all classes of consumers, the human and environmental health risks associated with inexpensive clothing are hidden throughout the lifecycle of each garment. From the growth of water-intensive cotton, to the release of untreated dyes into local water sources, to worker’s low wages and poor working conditions; the environmental and social costs involved in textile manufacturing are widespread.

In this paper, we posit that negative externalities at each step of the fast fashion supply chain have created a global environmental justice dilemma. While fast fashion offers consumers an opportunity to buy more clothes for less, those who work in or live near textile manufacturing facilities bear a disproportionate burden of environmental health hazards. Furthermore, increased consumption patterns have also created millions of tons of textile waste in landfills and unregulated settings. This is particularly applicable to low and middle-income countries (LMICs) as much of this waste ends up in second-hand clothing markets. These LMICs often lack the supports and resources necessary to develop and enforce environmental and occupational safeguards to protect human health. We discuss the role of industry, policymakers, consumers, and scientists in promoting sustainable production and ethical consumption in an equitable manner.

## Background

Fast fashion is a term used to describe the readily available, inexpensively made fashion of today. The word “fast” describes how quickly retailers can move designs from the catwalk to stores, keeping pace with constant demand for more and different styles. With the rise of globalization and growth of a global economy, supply chains have become international, shifting the growth of fibers, the manufacturing of textiles, and the construction of garments to areas with cheaper labor. Increased consumption drives the production of inexpensive clothing, and prices are kept down by outsourcing production to low and middle-income countries (LMICs).

Globally, 80 billion pieces of new clothing are purchased each year, translating to $1.2 trillion annually for the global fashion industry. The majority of these products are assembled in China and Bangladesh while the United States consumes more clothing and textiles than any other nation in the world [1]. Approximately 85 % of the clothing Americans consume, nearly 3.8 billion pounds annually, is sent to landfills as solid waste, amounting to nearly 80 pounds per American per year [2, 3].

The global health costs associated with the production of cheap clothing are substantial. While industrial disasters such as the 1911 Triangle Shirtwaist Factory fire have led to improved occupational protections and work standards in the United States, the same cannot be said for LMICs. The hazardous working conditions that attracted regulatory attention in the United States and European Union have not been eliminated, but merely shifted overseas. The social costs associated with the global textile and garment industry are significant as well. Defined as “all direct and indirect losses sustained by third persons or the general public as a result of unrestrained economic activities,” the social costs involved in the production of fast fashion include damages to the environment, human health, and human rights at each step along the production chain [4].

## Main text

### Fast fashion as a global environmental justice issue

Environmental justice is defined by the United States Environmental Protection Agency, as the “fair treatment and meaningful involvement of all people regardless of race, color national origin, or income, with respect to the development, implementation, and enforcement of environmental laws, regulations and policies” [5]. In the United States, this concept has primarily been used in the scientific literature and in practice to describe the disproportionate placement of superfund sites (hazardous waste sites) in or near communities of color. However, environmental justice, as it has been defined, is not limited to the United States and need not be constrained by geopolitical boundaries. The textile and garment industries, for example, shift the environmental and occupational burdens associated with mass production and disposal from high income countries to the under-resourced (e.g. low income, low-wage workers, women) communities in LMICs. Extending the environmental justice framework to encompass the disproportionate impact experienced by those who produce and dispose of our clothing is essential to understanding the magnitude of global injustice perpetuated through the consumption of cheap clothing. In the context of Sustainable Development Goal (SDG) 12 which calls for sustainable consumption and production as part of national and sectoral plans, sustainable business practices, consumer behavior, and the reduction and elimination of fast fashion should all be a target of global environmental justice advocates.

#### Environmental hazards during production

The first step in the global textile supply chain is textile production, the process by which both natural and synthetic fibers are made. Approximately 90 % of clothing sold in the United States is made with cotton or polyester, both associated with significant health impacts from the manufacturing and production processes [6]. Polyester, a synthetic textile, is derived from oil, while cotton requires large amounts of water and pesticides to grow. Textile dyeing results in additional hazards as untreated wastewater from dyes are often discharged into local water systems, releasing heavy metals and other toxicants that can adversely impact the health of animals in addition to nearby residents [6].

#### Occupational hazards during production

Garment assembly, the next step in the global textile supply chain, employs 40 million workers around the world [7]. LMICs produce 90% of the world’s clothing. Occupational and safety standards in these LMICs are often not enforced due to poor political infrastructure and organizational management [8]. The result is a myriad of occupational hazards, including respiratory hazards due to poor ventilation such as cotton dust and synthetic air particulates, and musculoskeletal hazards from repetitive motion tasks. The health hazards that prompted the creation of textile labor unions in the United States and the United Kingdom in the early 1900’s have now shifted to work settings in LMICs. In LMICs, reported health outcomes include debilitating and life-threatening conditions such as lung disease and cancer, damage to endocrine function, adverse reproductive and fetal outcomes, accidental injuries, overuse injuries and death [9–11]. Periodic reports of international disasters, such as the 2013 Rana Plaza factory collapse which killed 1134 Bangladeshi workers, are stark reminders of the health hazards faced by garment workers. These disasters, however, have not demonstrably changed safety standards for workers in LMICs [12].

#### Textile waste

While getting finished garments to consumers in the high-income countries is seen as the end of the line for the fashion industry, environmental injustices continue long after the garment is sold. The fast fashion model encourages consumers to view clothing as disposable. In fact, the average American throws away approximately 80 pounds of clothing and textiles annually, occupying nearly 5% of landfill space [3]. Clothing not sent directly to the landfill often ends up in the second-hand clothing trade. Approximately 500,000 tons of used clothing are exported abroad from the United States each year, the majority ending up in LMICs [8]. In 2015, the United States exported more than $700 million worth of used clothing [13]. Second-hand clothing not sold in the United States market is compressed into 1000-pound bales and exported overseas to be “graded” (sorted, categorized and re-baled) by low-wage workers in LMICs and sold in second-hand markets. Clothing not sold in markets becomes solid waste, clogging rivers, greenways, and parks, and creating the potential for additional environmental health hazards in LMICs lacking robust municipal waste systems.

### Solutions, innovation, and social justice

Ensuring environmental justice at each stage in the global supply chain remains a challenge. Global environmental justice will be dependent upon innovations in textile development, corporate sustainability, trade policy, and consumer habits.

#### Sustainable fibers

The sustainability of a fiber refers to the practices and policies that reduce environmental pollution and minimize the exploitation of people or natural resources in meeting lifestyle needs. Across the board, natural cellulosic and protein fibers are thought to be better for the environment and for human health, but in some cases manufactured fibers are thought to be more sustainable. Fabrics such as Lyocell, made from the cellulose of bamboo, are made in a closed loop production cycle in which 99% of the chemicals used to develop fabric fibers are recycled. The use of sustainable fibers will be key in minimizing the environmental impact of textile production.

#### Corporate sustainability

Oversight and certification organizations such as Fair Trade America and the National Council of Textiles Organization offer evaluation and auditing tools for fair trade and production standards. While some companies do elect to get certified in one or more of these independent accrediting programs, others are engaged in the process of “greenwashing.” Capitalizing on the emotional appeal of eco-friendly and fair trade goods, companies market their products as “green” without adhering to any criteria [14]. To combat these practices, industry-wide adoption of internationally recognized certification criteria should be adopted to encourage eco-friendly practices that promote health and safety across the supply chain.

#### Trade policy

While fair trade companies can attempt to compete with fast fashion retailers, markets for fair trade and eco-friendly textile manufacturing remain small, and ethically and environmentally sound supply chains are difficult and expensive to audit. High income countries can promote occupational safety and environmental health through trade policy and regulations. Although occupational and environmental regulations are often only enforceable within a country’s borders, there are several ways in which policymakers can mitigate the global environmental health hazards associated with fast fashion. The United States, for example, could increase import taxes for garments and textiles or place caps on annual weight or quantities imported from LMICs. At the other end of the clothing lifecycle, some LMICs have begun to regulate the import of used clothing. The United Nations Council for African Renewal, for example, recently released a report citing that “Rwanda, Tanzania and Uganda are raising taxes on secondhand clothes imports and at the same time offering incentives to local manufacturers” [15].

#### The role of the consumer

Trade policies and regulations will be the most effective solutions in bringing about large-scale change to the fast fashion industry. However, consumers in high income countries have a role to play in supporting companies and practices that minimize their negative impact on humans and the environment. While certifications attempt to raise industry standards, consumers must be aware of greenwashing and be critical in assessing which companies actually ensure a high level of standards versus those that make broad, sweeping claims about their social and sustainable practices [14]. The fast fashion model thrives on the idea of more for less, but the age-old adage “less in more” must be adopted by consumers if environmental justice issues in the fashion industry are to be addressed. The United Nation’s SDG 12, “Ensure sustainable consumption and production patterns,” seeks to redress the injustices caused by unfettered materialism. Consumers in high income countries can do their part to promote global environmental justice by buying high-quality clothing that lasts longer, shopping at second-hand stores, repairing clothing they already own, and purchasing from retailers with transparent supply chains.

## Conclusions

In the two decades since the fast fashion business model became the norm for big name fashion brands, increased demand for large amounts of inexpensive clothing has resulted in environmental and social degradation along each step of the supply chain. The environmental and human health consequences of fast fashion have largely been missing from the scientific literature, research, and discussions surrounding environmental justice. The breadth and depth of social and environmental abuses in fast fashion warrants its classification as an issue of global environmental justice.

Environmental health scientists play a key role in supporting evidence-based public health. Similar to historical cases of environmental injustice in the United States, the unequal distribution of environmental exposures disproportionally impact communities in LMICs. There is an emerging need for research that examines the adverse health outcomes associated with fast fashion at each stage of the supply chain and post-consumer process, particularly in LMICs. Advancing work in this area will inform the translation of research findings to public health policies and practices that lead to sustainable production and ethical consumption.

## Abbreviations

LMICs: Low and middle-income countries
SDG: Sustainable Development Goal
